# Biomarkers of Broccoli Consumption: Implications for Glutathione Metabolism and Liver Health

**DOI:** 10.3390/nu12092514

**Published:** 2020-08-20

**Authors:** Alicia Arredondo Eve, Xiaoji Liu, Yanling Wang, Michael J. Miller, Elizabeth H. Jeffery, Zeynep Madak-Erdogan

**Affiliations:** 1 Department of Food Science and Human Nutrition, University of Illinois, Urbana-Champaign, Urbana, IL 61801, USA; aliciaa2@illinois.edu (A.A.E.); xiaoji2@ualberta.ca (X.L.); ywang153@gsu.edu (Y.W.); mille216@illinois.edu (M.J.M.); ejeffery@illinois.edu (E.H.J.); 2 Division of Nutritional Science, University of Illinois, Urbana-Champaign, Urbana, IL 61801, USA; 3 Carl R. Woese Institute of Genomic Biology, University of Illinois, Urbana-Champaign, Urbana, IL 61801, USA; 4 Cancer Center at Illinois, University of Illinois, Urbana-Champaign, Urbana, IL 61801, USA; 5 Beckman Institute for Advanced Science and Technology, University of Illinois, Urbana-Champaign, Urbana, IL 61801, USA

**Keywords:** broccoli, metabolic circulating markers, liver

## Abstract

Diet and lifestyle choices contribute to obesity and liver disease. Broccoli, a brassica vegetable, may mitigate negative effects of both diet and lifestyle. Currently, there are no clinically relevant, established molecular biomarkers that reflect variability in human absorption of brassica bioactives, which may be the cause of variability/inconsistencies in health benefits in the human population. Here, we focused on the plasma metabolite profile and composition of the gut microbiome in rats, a relatively homogenous population in terms of gut microbiota, genetics, sex and diet, to determine if changes in the plasma metabolite profiles caused by dietary broccoli relate to molecular changes in liver. Our aim was to identify plasma indicators that reflect how liver health is impacted by dietary broccoli. Rats were fed a 10% broccoli diet for 14 days. We examined the plasma metabolite composition by metabolomics analysis using GC–MS and gut microbiota using 16S sequencing after 0, 1, 2, 4, 7, 14 days of broccoli feeding. We identified 25 plasma metabolites that changed with broccoli consumption, including metabolites associated with hepatic glutathione synthesis, and with de novo fatty acid synthesis. Glutamine, stearic acid, and S-methyl-L-cysteine (SMC) relative abundance changes correlated with changes in gut bacteria previously implicated in metabolic disease and with validated increases in expression of hepatic NAD(P)H dehydrogenase [quinone] 1 (NQO1) and nuclear factor (erythroid-derived 2)-like 2 (Nrf2), associated with elevated hepatic glutathione synthesis. Circulating biomarkers following broccoli consumption reflect gut–liver axis health.

## 1. Introduction

The sale of broccoli, a brassica vegetable, has shown substantial growth in the food market over the past 20 years [1,2,3], due, in part, to news of health promotion associated with dietary broccoli. Epidemiological studies report that incorporating brassica into our daily diets can decrease cancer risk at several sites, including bladder, breast, prostate, and colon [4,5,6,7]. In addition, dietary broccoli impedes the development of non-alcoholic fatty liver disease (NAFLD) and hepatic triglyceride accumulation in mice fed a Western diet [8], suggesting that dietary broccoli can play a role in maintaining liver health, important in a society with a high rate of obesity and no good drugs to fight NAFLD. Yet, because there are also a number of clinical studies that show no benefit of dietary broccoli, advisory bodies such as the American Institute for Cancer Research rarely promote the health benefits of broccoli. The cause of this variability in findings may relate to the dose consumed and fraction of sulforaphane (SF) absorbed from a single serving, which can vary 20-fold or more; the differences in preparation and freshness, which impact the glucoraphanin (GRP) hydrolyzing enzyme myrosinase [9]; and variation in intestinal microbiota (IM) composition of the subjects [10], which is responsible for ITC production when cooked broccoli is consumed.

Broccoli secondary metabolites, the glucosinolates (GSL), are hydrolyzed to bioavailable products, including bioactive isothiocyanates (ITC), by either plant myrosinase or gut microbial (IM) enzymes [2,11]. Previous studies show that as few as four days’ broccoli feeding changed the rat fecal microbiota diversity by increasing the abundance of *Bacteroides/Prevotella/Porphyromonas* and decreasing the abundance of pathogenic *Clostridium perfringens* [12]. Additionally, long-term dietary broccoli alters IM diversity in humans and rats [10,13]. Unfortunately, very little is known about which gut microorganisms are responsible for hydrolyzing GSLs to ITCs. The variability in the IM across individuals contributes particularly to the variation in ITC absorption when cooked brassica (myrosinase inactivated) is consumed [14]. Previously, we demonstrated that when rats consume cooked broccoli daily for 4 days or more, this alters the IM, increasing the daily ITC produced by the IM, since without active myrosinase, more GSL passes into the colon [11].

Chronic inflammation, disrupted lipid homeostasis, oxidative stress, imbalance of IM and increased endotoxin exposure of hepatocytes due to a leaky gut may all play a part in causing lipid accumulation in liver of obese individuals [15,16]. Dietary broccoli prevents the development of NAFLD and hepatic triglyceride accumulation in mice fed a high-fat diet, reflective of the typical American ‘Western Diet’ [8]. These data suggest that dietary broccoli may play a role in maintaining liver health, important in a society with a high rate of obesity, type 2 diabetes and NAFLD [8].

We still lack clinical data definitively showing the health benefit of dietary broccoli, due in part to variability in the fraction of GRP hydrolyzed and thus of SF absorbed, in preparation and freshness of broccoli, and in IM makeup of the subjects [8,10]. Even though urinary metabolites are used to track broccoli consumption, these metabolite biomarkers do not fully reflect bioavailability of ITC, or the fraction that reaches target tissues. Reliable biomarkers of SF production, absorption, and activity could clarify inconsistent data and allow a more accurate determination of the health benefit of broccoli consumption. Use of rodents, which are relatively homogenous in their IM biology and dietary responses, can provide tissue samples such as liver, and are good model systems for metabolic health that have been proven to be useful in identifying biomarkers [17]. Thus, our goal is to establish circulating biomarkers of long-term broccoli consumption, their association with changes in the IM and document if such changes reflect activation of health-beneficial pathways in the liver.

## 2. Materials and Methods

### 2.1. Animals and Diets

Animal use was approved by the Illinois Institutional Animal Care and Use Committee, according to National Institutes of Health guidelines (Protocol # 18033). Fischer 344 rats weighing 120–140 grams were purchased from Harlan (Colony 217, Indianapolis, IN, USA; Colony 208, Frederick, MD, USA) and housed individually in cages, given food and water ad libitum. Rats were acclimated to a powdered AIN93G diet [11] for five days before transitioning to experimental diets. Commercially available frozen cooked broccoli (CB) was freeze-dried before incorporation into the diets. Broccoli diets were balanced to provide similar macronutrient content to the AIN93G diet, according to the USDA National nutrient database [18]. The CB was confirmed free of both myrosinase activity and SFN, but with 0.11 mg/g diet GRP [11]. All rats were anesthetized with ketamine/xylazine (87 and 13 mg/mL, respectively; 1 mL/100 g BW), blood drawn, and then killed by cervical dislocation. The ceca were ligated anteriorly and distally, surgically removed, and immediately transferred to an anaerobic chamber to remove the microbiome for DNA isolation.

### 2.2. Long-Term Broccoli Feeding Study

**Figure 1:**
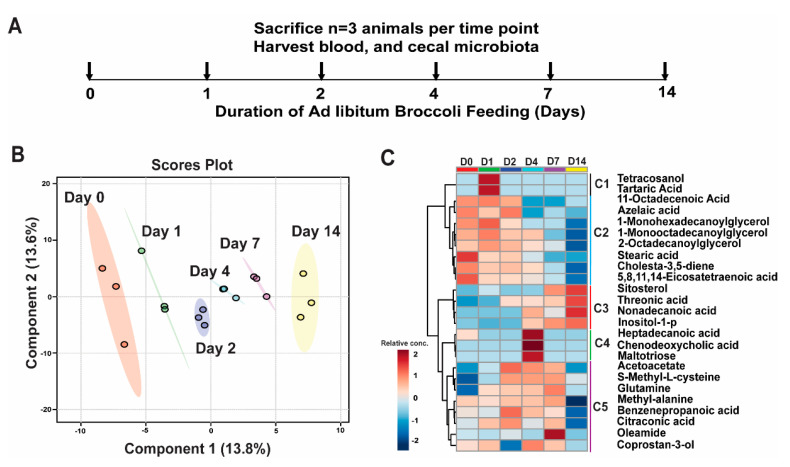
Broccoli feeding alters plasma metabolite composition (**A**) Experimental timeline (**B**) PLSDA plot of plasma metabolite composition. (**C**) Hierarchical clustering of the top 25 metabolites that contributes to PLSDA plot in 1A. D0 = Day 0.

In study one, 18 rats (Colony 208) were acclimated to AIN93G (control; CON) diet, randomized to six groups of three, then fed the CB diet ad libitum for 0, 1, 2, 4, 7, or 14 days (Figure 1A). Three rats were sacrificed at each time point and blood was collected from the abdominal aorta. The study was then repeated for protein expression analysis in liver. In this second study, to examine the effects of broccoli feeding in liver protein expression, eight rats (Colony 217) were divided into two groups of four, and were fed (1) CON or (2) CB diet for four days. Variation in broccoli, such as due to presence of different amounts of epithiospecifier protein, are excluded during the experiment, where all animals consumed divided samples of the same broccoli preparation.

### 2.3. Cecal Microbiota Profiling by 16S rRNA Sequencing

Total DNA was extracted from 40 mg of cecal contents using QIAmp DNA stool Mini Kit (Qiagen, Alameda, CA, USA) with bead-beating [19,20]. Concentration and quality of DNA were measured using NanoDrop (Thermo Scientific, Franklin, MA, USA). The 16S rRNA sequencing was performed by the Roy J. Carver Biotechnology Center, University of Illinois at Urbana-Champaign (RJC/UIUC). The V3-V5 variable regions of the 16S rDNA were amplified using primers (forward F357: 5 0 -CCTACGGGAGGCAGCAG-30 and reverse R926: 50 -CCGTCAATTCMTTTRAGT-30). The quality of the PCR products was analyzed using an Agilent 2100 BioAnalyzer. Paired-end reads were generated with the Illumina^®^ MiSeq platform for each sample, at a read length of 250 nucleotides. The reads were demultiplexed and quality-filtered using Trimmomatic [21] (version 0.30, USADELLAB.org, RRID:SCR_011848, Worringerweg, Aachen, Germany) with a Phred score cut-off of 33. Data analysis was performed through the QIIME pipeline including chimera removal (version 1.9.0, RRID:SCR_008249, Scikit-bio™, Boulder, CO, USA). Operational taxonomical unit (OTU)-picking was performed by searching the Greengenes 16S rRNA gene database [22].

### 2.4. Metabolic Profiling

Whole blood (500 µL) was extracted from the abdominal aorta at the time of tissue harvest. Ten μL of 0.5 M EDTA was added to blood samples and samples were centrifuged for 10 min at room temperature at 1000× *g*. Supernatant (plasma) was removed and stored at −80 °C. Plasma samples were submitted to the Metabolomics Center at the RJC/CBC. Gas chromatography–mass spectrometry (GC/MS) whole metabolite profiling was performed to detect and quantify the metabolites by using GC/MS analysis as described [23]. Briefly, 50 μL of blood plasma was extracted using 1 mL of isopropanol:acetonitrile:water (3:3:2, *v/v*) at 20 °C for 5 min. After centrifugation, 0.5 mL of supernatant was dried in a SpeedVac concentrator and subsequently derivatized in two steps: with 50 μL methoxyamine hydrochloride (Sigma-Aldrich, St. Louis, MO, USA) (40 mg/mL in pyridine) for 60 min at 50 °C, then with 50 μL MSTFA + 1%TMCS (Thermo, MA, USA) at 70 °C for 120 min, followed by a 2 h incubation at room temperature. Hentriacontanoic acid (30 μL of 1 mg/mL) was added to each sample prior to derivatization for use as an internal standard for normalization. Metabolite profiles were acquired using a gas-chromatography mass-spectrometry (GC-MS) system (Agilent Inc., Santa Clara, CA, USA) consisting of an Agilent 7890 gas chromatograph, an Agilent 5975 MSD and 7683B autosampler, as previously described [24]. Briefly, gas chromatography was performed on a ZB-5MS (60 m × 0.32 mm I.D. and 0.25 mm film thickness) capillary column (Phenomenex, Torrance, CA, USA). The inlet and MS interface temperatures were 250 °C, and the ion source temperature was adjusted to 230 °C. An aliquot of 1 mL was injected with the split ratio of 10:1. The helium carrier gas was kept at a constant flow rate of 2.4 mL/min. The temperature program was: 5 min isothermal heating at 70 °C, followed by an oven temperature increase of 5 °C/min to °C after which a final 10 min incubation at 310 °C was performed. The mass spectrometer was operated in positive electron impact mode (EI) at 69.9 eV ionization energy at m/z 30–800 scan range. The scan range was set at least 50 m/z above the highest anticipated fragment. The minimum quality match for minor compounds was ≥80 and for other peaks ≥90. The spectra of all chromatogram peaks were evaluated using the AMDIS 2.71 (NIST, Gaithersburg, MD, USA) using a custom-built MS database (484 unique metabolites) [24]. Tentative substances were not reported. The identified metabolites list was analyzed using MetaboAnalyst (version 4.0, RRID:SCR_015539). Using statistical analysis, including partial least-squares discriminant analysis (PLSDA), and a heatmap, which featured 25 metabolites generated using default options for clustering and restricting the data to the top 25 metabolites ranked by *t*-test. Differences in metabolite profiles among day 1, 2, 4, 7 and 14 were analyzed using Prism 7.0 (GraphPad software, San Diego, CA, USA, RRID:SCR_002798) with two-tailed *t*-tests followed by two-way-ANOVA. *p* < 0.05 was considered significant. Pathway analysis was also carried out using differences among these metabolite profiles between broccoli feeding days using Metabolite Set Enrichment Analysis to identify Pathway-associated metabolite sets (SMPDB) analysis (Metaboanalyst 4.0).

### 2.5. Western Blot Analysis of Hepatic Protein Expression

Total protein was isolated from liver by homogenization in lysis buffer (0.5 M EDTA, 1 M Tris-HCl pH 8.1, 10% SDS, 10% Empigen,) with 1 × Complete Protease Inhibitor (Roche) and 1 × Phosphatase Inhibitor (Thermo Scientific, Waltham, MA, USA). Samples were further processed with sonication and protein concentrations were determined (BCA assay, Thermo Scientific). Supernatants were collected and filtered using an Amicon Ultra-4 centrifugal filter device (Millipore Sigma) after Western blot analysis. Equal amounts of protein were separated by electrophoresis on 4–20% Mini-PROTEAN TGX gels (BIORAD) and transferred to nitrocellulose membranes. Membranes were blocked (Blocking Buffer, Odyssey^®^, Li-Cor, Lincoln, NE, USA) and target proteins were probed with the following primary antibodies: β-actin (Sigma SAB1305546) 1:10,000 dilution, NAD(P)H dehydrogenase [quinone] 1 (NQO1) (Abcam Cat# ab2346, RRID:AB_302995) 1:500 dilution, nuclear factor (erythroid-derived 2)-like 2 (Nrf2) (R&D SYSTEM MAB3925) 1:500 dilution. Membranes were then exposed to an anti-goat, anti-mouse secondary antibody (Odyssey) at 1:10,000 dilution. Proteins were visualized using a Licor Odyssey CLx infrared imaging device and software. All results were repeated at least three times and the results were normalized to a β-actin loading control.

### 2.6. Statistical Analysis

For both the plasma metabolomic profile and the cecal IM, a two-way-ANOVA model (Dunnett’s multiple comparison test) was used to compare time-dependent changes. For IM and plasma metabolite correlation, simple linear regression was used. All normally distributed data were analyzed using pairwise *t*-tests with a Bonferroni correction to identify treatments that were different from each other (*p* < 0.05, *p* < 0.01, *p* < 0.001, *p* < 0.0001). For each main effect that was significant at α = 0.05, pairwise *t*-tests were conducted to determine if the diet induced significant changes in the level of metabolites. For those *t*-tests, the Bonferroni correction was employed to control experiment-wise type I error rate at α = 0.05, followed by Bonferroni post hoc test. Data that were not normally distributed were analyzed using the Mann–Whitney test for nonparametric data (*p* < 0.05, *p* < 0.01, *p* < 0.001, *p* < 0.0001).

For Western Blot analysis, unpaired *t*-tests was used to compare different diet effects. For every main effect that was significant at *p* < 0.05, pairwise *t*-tests were conducted to determine if the diet affected the expression of Nrf2 or NQO1. For those *t*-tests, the Bonferroni correction was employed to control the experiment-wise type I error rate at α = 0.05, followed by Bonferroni post hoc test using GraphPad Prism 7.0 (RRID: SCR_002798). All values for all the experiments were plotted.

## 3. Results

### 3.1. Broccoli Feeding Alters Plasma Metabolite Profile

To identify broccoli-dependent changes in the compositional profile of plasma metabolites, plasma was obtained from three rats fed AIN93G diet with 10% freeze-dried CB daily for 0, 1, 2, 4, 7 or 14 days (Figure 1A) and a metabolomics analysis was performed using GC–MS. Of the 184 metabolites identified (Supplemental Table S1), content of 21 metabolites were altered significantly (2-way ANOVA, time course model, post-test Dunnet’s multiple comparison *p* < 0.05) by broccoli feeding (Table 1).

Using the statistical analysis tool in web-based Metaboanalyst software, we assessed changes in the relative concentration of metabolites for increasing days of broccoli feeding (Figure 1A). 2D PLSDA analysis showed that plasma samples clustered based on the number of days rats were fed broccoli (Figure 1B). Clustering of the top 25 metabolites that contributed to separation of samples in PLSDA analysis showed that metabolites separated into five clusters based on their changing pattern through the broccoli feeding period (C1 through C5). Cluster C1 contained metabolites tetracosanol and tartaric acid levels increased on day 1 (D1) of broccoli feeding. Cluster C2 metabolites included 11-octadecenoic acid, azelaic acid, 1-monohexadecanoylglycerol, 1-monooctadecanoylglycerol, 2-octadecanoylglycerol, stearic acid, cholesta-3, 5-diene, 5, 8, 11, 14-eicosatetraenoic acid; these decreased slowly throughout the broccoli feeding (Figure 1B,C). In cluster C3, sitosterol, threonic acid, nonadecanoic acid and inositol-1-phosphate, were the main metabolites to increase throughout the 14 days of broccoli feeding. Cluster C4 contained metabolites heptadecanoic acid, chenodeoxycholic acid and maltotriose, and their relative concentration was only increased on day 4 (D4) of the broccoli feeding. Finally, cluster C5 contained metabolites including acetoacetate, SMC, glutamine, methyl-alanine, benzenepropanoic acid, citraconic acid, oleamide and coprostan-3-ol; plasma levels of these metabolites increased on days 2, 4 and 7 (D2, D4, and D7), but dropped again by day 14. These top 25 metabolites were chosen for focus because of the significant changes in abundance, compared to abundance on day 0.

**Figure 2:**
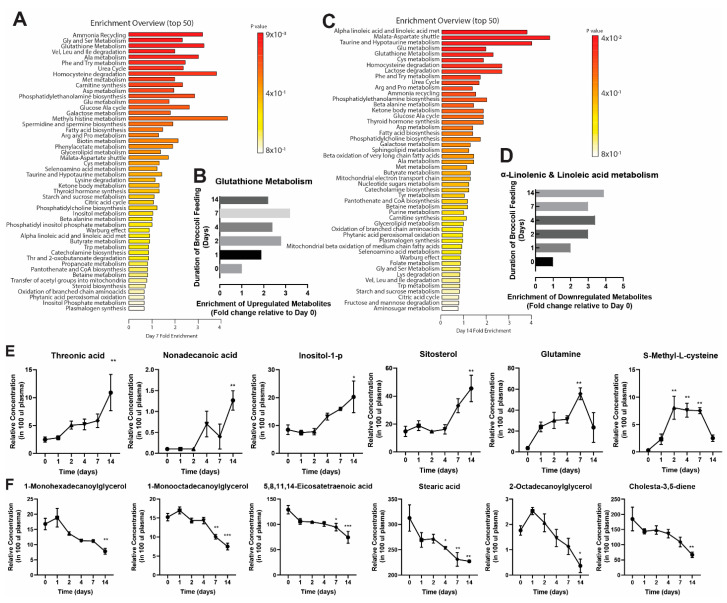
Pathway analysis of metabolite enrichment of up- (**A**,**B**) and down-regulated (**C**,**D**) metabolites. Relative concentration of up- (**E**) and down-regulated (**F**) metabolites that change significantly over 14 days of broccoli feeding.

To identify which metabolic pathways were overrepresented in each cluster, we utilized the pathway analysis tool in Metaboanalyst (Figure 2A,C). This analysis showed that amino acid synthesis and glutathione (GSH) synthesis pathways were upregulated by Day 7 (Figure 2A,B) and fatty acid synthesis pathways, specifically α-linoleic acid synthesis pathways were downregulated by Day 14 (Figure 2C,D). We identified twelve metabolites (Figure 2E,F), several of which were shown to be associated with the GSH status (glutamine, 0 vs. 7 days, *p* = 0.0014; S-methyl-L-cysteine, 0 vs. 2 days, *p* = 0.0016; 0 vs. 4 days, *p* = 0.0026; and 0 vs. 7 days, *p* = 0.0028), de novo fatty acid synthesis pathway (nonadecanoic acid, 0 vs. 14 days, *p* = 0.0056; 1-monohexadecanoylglycerol, 0 vs. 14 days, *p* = 0.0060; 11-octadecenoic acid, 0 vs. 4 days, *p* = 0.0124; 0 vs. 7 days, *p* = 0.0143; 5,8,11,14-eicosatetraenoic acid, 0 vs. 7 days, *p* = 0.0227; 0 vs. 14 days, *p* = 0.0008; stearic acid, 0 vs. 4 days, *p* = 0.0398; 0 vs. 7 days, *p* = 0.0053; 0 vs. 14 days, *p* = 0.0038; 2-octadecanoylglycerol, 0 vs. 14 days, *p* = 0.0159; cholesta-3,5-diene, 0 vs. 14 days, *p* = 0.0046; inositol-1-p, 0 vs. 14, *p* = 0.0264) and amino acid synthesis pathways (threonic acid, 0 vs. 14, *p* = 0.0080) (Figure 2C,D). In summary, our data show that broccoli consumption changes the abundance of metabolites related to several different metabolic pathways.

### 3.2. Changes in Gut Microbiota Composition Correlate with Changes in Plasma Metabolites Following Long-Term Broccoli Feeding

The time course for the change in rat cecal microbiota composition was profiled using 16S rRNA sequencing (*n* = 3, each time point) to determine changes in bacterial taxa abundance following broccoli consumption from 0 to 14 days. As previously reported, continuous broccoli feeding alters the cecal bacterial composition in rats [11]. Using the statistical analysis tool in the web-based MicrobiomeAnalyst software, we observed that some bacteria from the phyla Firmicutes, Bacteroidetes, Actinobacteria, and Proteobacteria changed significantly (*p* < 0.05) over 14 days of broccoli feeding (Table 2).

**Figure 3:**
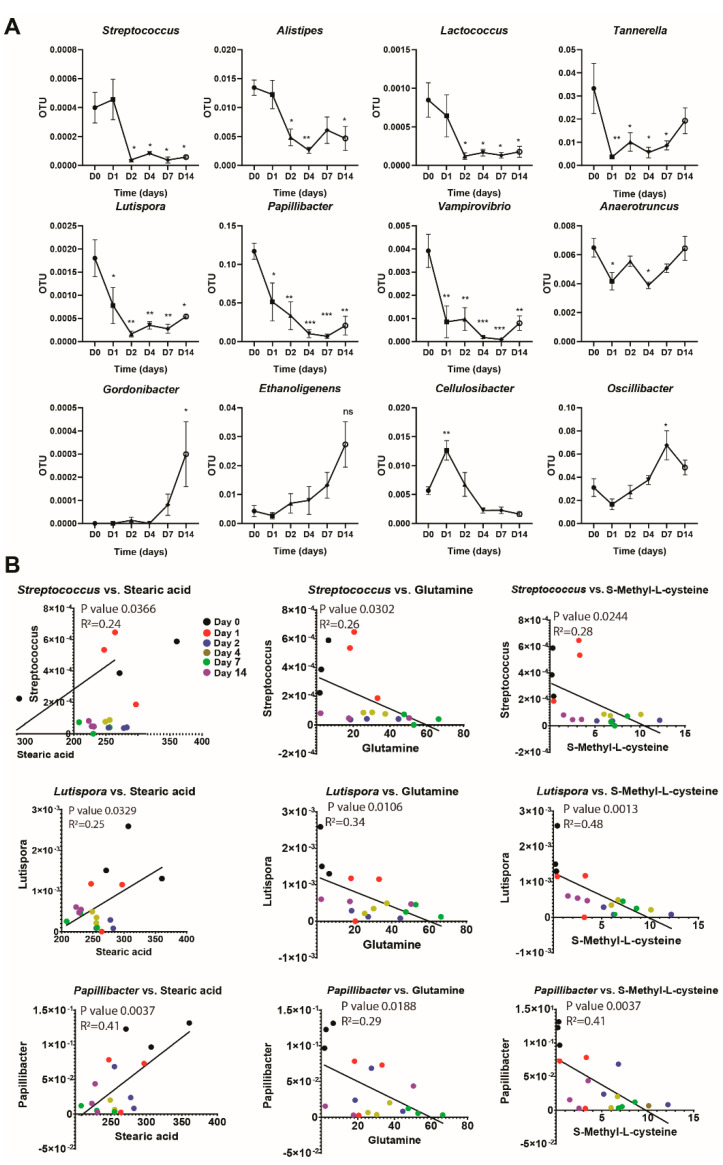
Changes of gut microbiota in relation to metabolite changes after broccoli feeding. (**A**) Time course of OTU change for bacteria. Unpaired *t*-test, *Firmicutes*-*Lactobacillales*—*Streptococcus*, 0 vs. 2 days, *p* = 0.0202; 0 vs. 4 days, *p* = 0.0440; 0 vs. 7 days, *p* = 0.0196 and 0 vs. 14 days, *p* = 0.0282; *Bacteroidetes-Bacteroidales*—*Alistipes*, 0 vs. 1 day, *p* = 0.0220; 0 vs. 4 days, *p* = 0.0047; and 0 vs. 14 days, *p* = 0.0196; *Firmicutes-Lactobacillales—Lactococcus*, 0 vs. 2 days, *p* = 0.0190; 0 vs. 4 days, *p* = 0.0279; 0 vs. 7 days, *p* = 0.0209 and 0 vs. 14 days, *p* = 0.0306; *Bacteroidetes-Bacteroidales-Tannerella*, 0 vs. 1 day, *p* = 0.0088; 0 vs. 2 days, *p* = 0.0400; 0 vs. 4 days, *p* = 0.0137 and 0 vs. 7 days, *p* = 0.0282; *Firmicutes-Clostridiales—Lutispora*, 0 vs. 1 day, *p* = 0.0370; 0 vs. 2 days, *p* = 0.0015; 0 vs. 4 days, *p* = 0.0039, 0 vs. 7 days, *p* = 0.0026 and 0 vs. 14 days, *p* = 0.0104; *Firmicutes-Clostridiales-Ruminococcaceae*—*Papillibacter*, 0 vs. 1 day, *p* = 0.0262; 0 vs. 2 days, *p* = 0.0055; 0 vs. 4 days, *p* = 0.0008, 0 vs. 7 days, *p* = 0.0006, and 0 vs. 14 days, *p* = 0.0018; *Proteobacteria-Bdellovibrionale*—*Vampirovibrio*, 0 vs. 1 day, *p* = 0.0025; 0 vs. 2 days, *p* = 0.0034; 0 vs. 4 days, *p* = 0.0005, 0 vs. 7 days, *p* = 0.0004, and 0 vs. 14 days, *p* = 0.0022, *Actinobacteria-Coriobacteriaceae-Gordonibacter*, 0 vs. 14 days, *p =* 0.0172; *Firmicutes-Clostridiales-Ruminococcaceae—Ethanoligenens*, 0 vs. 14 days, *p* = 0.0138; *Firmicutes-Clostridiales-Ruminococcaceae-Cellulosibacter*, 0 vs. 1 day, *p* = 0.0049; *Firmicutes-Clostridiales-Ruminococcaceae—Oscillibacter*, 0 vs. 7 days, *p* = 0.0173 (**B**) Correlation of relative concentration of metabolites and bacteria that changed significantly over 14 days of broccoli feeding.* *p* < 0.05, ** *p* < 0.01, *** *p* < 0.001.

The pattern of changes in bacteria was variable (Figure 3A). Streptococcus, Alistipes, Lactococcus, Tannerella, Lutispora, Papillibacter, and Vampirovibrio decreased throughout the feeding and stayed decreased. Anaerotruncus decreased but returned to normal at the end of the 14 days (0 vs. 1 days, *p* = 0.0380 and 0 vs. 4 days, *p* = 0.0195). Gordonibacter and Ethanoligenens increased during the study and stayed increased. Cellulosibacter and Oscillibacter increased initially but returned to normal at the end of the 14 days (Figure 3A). Using Pearson’s correlation analysis, we identified several genera from the phylum Firmicutes, such as Streptococcus, Lutispora, and Papillibacter, to be highly correlated with three of the plasma metabolites of interest; glutamine, stearic acid, and SMC (Figure 3B). Stearic acid exhibited a positive correlation with all three of these bacterial genera (Streptococcus, *p* = 0.0244; Lutispora, *p* = 0.0013; Papillibacter, *p* = 0.0037). In contrast, a negative correlation was seen for the same bacteria with glutamine (Streptococcus, *p* = 0.0302; Lutispora, *p* = 0.0106; Papillibacter, *p* = 0.0188) and SMC (Streptococcus, *p* = 0.0244; Lutispora, *p* = 0.0013; Papillibacter, *p* = 0.0037). In summary, our data show a correlation between the abundance of certain gut bacteria and levels of certain circulating plasma metabolites after long-term broccoli feeding.

### 3.3. Broccoli Feeding Increases Protein Expression of NAD(P)H Dehydrogenase [Quinone] 1 (NQO1) and Nuclear Factor (Erythroid-Derived 2)-like 2 (Nrf2) in Rat Liver

**Figure 4:**
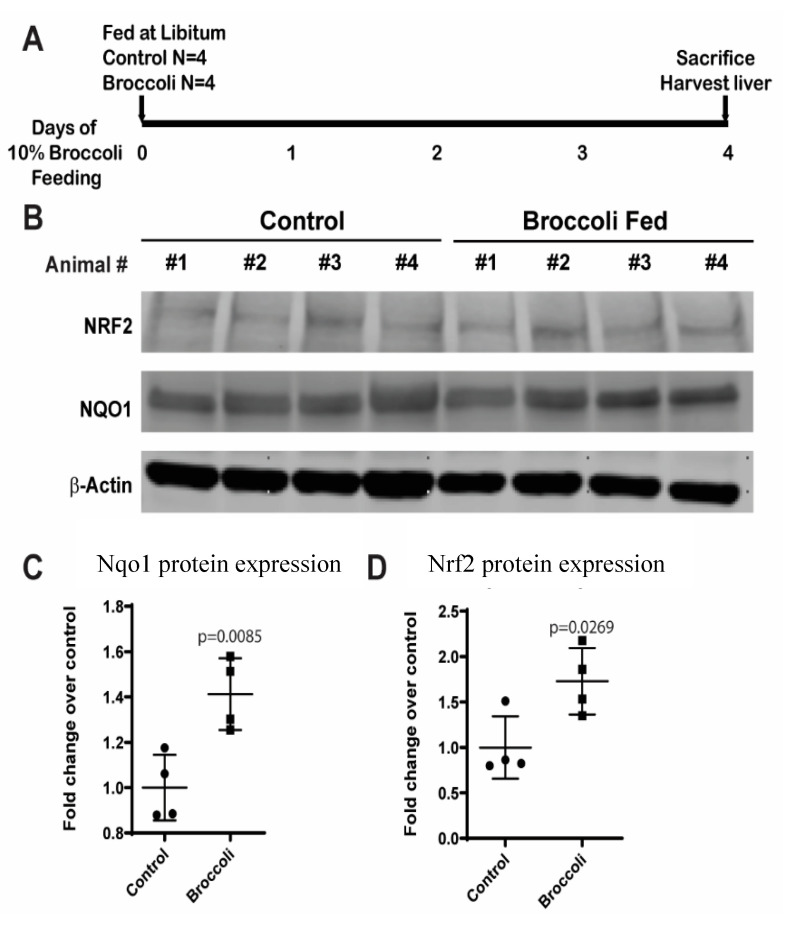
Broccoli feeding increases protein expression of Nqo1 and Nrf2 in rats after 14 days of broccoli feeding (**A**) Timeline of study. Rats were fed a diet with or without 10% freeze-dried broccoli (*n* = 4/feeding group). (**B**) The protein expression of Nqo1 and Nrf2 in liver tissue was detected with Western blot. β-actin served as a loading control, used to normalize Nqo1 and Nrf2 protein expression and calculate data for C,D. (**C**) Quantification of Western blot results. Unpaired *t*-test analysis of Nqo1(**C**) and Nrf2 (**D**) protein expression. *p* < 0.05 as compared with the control group, all data and standard deviation are plotted.

Having observed changes in plasma metabolite abundance associated with GSH pathways, we monitored hepatic expression of the GSH synthesis trigger protein, Nrf2; since liver is the main site of GSH synthesis. Nrf2 activates genes that bear an antioxidant response element (ARE), including γ-glutamyl cysteine ligase (GCL), the regulatory enzyme for GSH synthesis [25]. To validate the impact of broccoli consumption on Nrf2 in the liver, we performed Western blot analysis for both the Nrf2 and Nrf2 target, Nqo1 (Figure 4B). Nqo1 and Nrf2 protein content were quantified in liver samples from four control and four 4-day broccoli-fed rats. The Nqo1 protein was increased 1.5-fold in livers of broccoli-fed rats compared to controls (Unpaired *t*-test, *p* = 0.0085). For Nrf2, the protein concentration was increased 1.7-fold by broccoli feeding (Unpaired *t*-test, *p* = 0.0269) (Figure 4C). In summary, rats fed a broccoli diet exhibit an enhanced Nrf2-Nqo1 pathway by day 4.

## 4. Discussion

Numerous rodent and clinical studies support the appearance of health benefits in individuals who eat broccoli frequently, yet there are also clinical studies that show little or no benefit from dietary broccoli. Biomarkers of health promotion by broccoli consumption are not established, but are needed if we are to understand human variability in response. Here, we took advantage of a homogenous population of rats and focused on plasma metabolite changes that might be developed as biomarkers for a healthy liver and evaluated whether this might also relate to changes in the IM upon broccoli feeding for 4 days or more. Interestingly, our metabolomics screen identified metabolites related to GSH synthesis and turnover. Broccoli feeding is well known to cause a temporary loss of hepatic GSH followed by enhanced synthesis [26]. As a secondary interest, we had earlier noted a reversal of fatty liver in broccoli-fed mice [8], although in that study we did not evaluate change in plasma fatty acids that might act as biomarkers of NAFLD status and liver health. Thus, a secondary hypothesis here was that any change in hepatic lipid storage related to broccoli feeding might be reflected in plasma changes that could act as biomarkers—both for broccoli efficacy and unrelated to broccoli, for biomarkers of NAFLD status. For example, other brassica might trigger some of the same biomarkers to appear, or even other foods known to positively impact hepatic health might do this.

Here, we report that, given that a key impact of broccoli consumption is that it is known to enhance hepatic GSH and resulting anti-oxidative effects, we were interested to see that the concentration of two plasma metabolites associated with hepatic GSH metabolism, glutamine and SMC, were among those metabolites in cluster 2, seen to consistently increase with daily broccoli consumption (Figure 1). Reviewing the literature, we found earlier instances where these two metabolites had been associated with changes in hepatic GSH status, again supporting the possibility of their acting as biomarkers of the impact of broccoli feeding on liver health. In one study, rats were given fructose with or without SMC, and SMC was associated with protection against oxidative stress, normalized hepatic GSH levels and diminished type 2 diabetes [27]. In another study, dietary glutamine prevented carbon tetrachloride (CCl_4_)-induced liver fibrosis in mice, reversing many of the CCl_4_ effects in liver (elevated serum alanine aminotransferase and bilirubin, decreased albumin and increased liver collagen deposition) [28]. Whereas a biochemical relationship between plasma biomarkers and liver health is not necessarily expected for biomarkers, it is exciting to note that these two potential biomarkers are clearly associated with pathways known to be impacted by brassica consumption. Furthermore, since we know that the GSL metabolite SF alters liver GSH metabolism, it might be that consumption of any brassica, since all have GSLs, may lead to plasma glutamine and SMC as biomarkers. Future studies are needed to confirm whether glutamine and SMC are broccoli-specific or GSL-specific biomarkers.

Previously, a small clinical study reported changes in plasma fatty acids and GSH/GSH component levels after even a single meal of broccoli sprouts, similar to pathways we report here for rat plasma [29]. Differences in findings might be due to the shorter duration of the clinical study. Reviewing our data on components associated with GSH synthesis, we saw levels drop initially, then rise. In Housley’s study, lasting only 2 days, levels dropped like ours, but the study was not sufficiently long to see the recovery and overshoot that we saw by 14 days when glutamine abundance and liver Nrf2 and NQO1 expression were all increased, suggesting increased GSH production, which might provide protection of liver from reactive oxygen species (ROS).

Plasma concentration of several metabolites important to fatty acid metabolism were lower in broccoli-fed rats. Our findings that relate to fatty acid synthetic pathways are also consistent with several clinical studies, suggesting that the long-term broccoli consumption biomarkers that we identified here in rodents are relevant to humans. One of the metabolites that was decreased over the 14 days of broccoli feeding was 11-octadecenoic acid, involved in de novo lipid synthesis and deposition in liver [30]. Past studies report that 11-octadecenoic acid levels correlate across plasma and liver, as well as with NAFLD progression, supporting potential future use of a decreased plasma level of 11-octadecenoic acid as an indicator of broccoli consumption and/or lessened NAFLD and improved liver health [31,32]. A similar pattern was seen for stearic acid, levels of which were previously shown to correlate with dietary lipid intake, insulin resistance and advanced liver fibrosis [33]. On the other hand, neither sitosterol nor conjugated linoleic acid were identified as potential biomarkers here, even though they have been reported to correlate with improved metabolic health including low plasma cholesterol, low body weight gain, low hepatic lipid accumulation, and alleviated NAFLD in mice [34,35], factors that are increased with broccoli consumption (8). Overall, these findings support the liver health benefits of long-term broccoli consumption and validate several metabolites as indicators of beneficial effects of broccoli consumption on lipid normalization. There is presently no easy clinical measure of NAFLD—and plasma 11-octadecenoic acid or other fatty acids may provide a much-needed biomarker.

In this rodent study, we find that within the phylum *Firmicutes,* dietary broccoli caused changes in three different genera, *Streptococcus*, *Lutispora*, and *Papillibacter*, and that this change correlated with changes in three plasma metabolites, stearic acid, glutamine and SMC (Figure 2). Stearic acid correlated positively with all three genera (less stearic acid, fewer bacteria, at the end of the 14 days). In contrast, diet-induced changes in plasma glutamine and SMC were negatively correlated with broccoli-dependent changes in these three genera (fewer bacteria more metabolites) (Figure 2). This finding is similar to that of a study where the aim was to determine the effects of dietary supplementation with 1% L-glutamine for 14 days, on the abundance of IM. They reported that glutamine supplementation decreased the abundance of *Firmicutes* [35]. This supports our findings, negatively correlating cecal *Firmicutes* abundance with plasma glutamine over the 14 days of broccoli feeding. In contrast, we found no correlation with *Bacteroides*, despite the recent discovery of an IM *Bacteroides thetaiotaomicron* strain that can convert GSLs to ITCs. This is likely due to the rare frequency of this activity amongst *Bacteroides*. More work is needed to identify the microbes responsible for ITC production in rat and human gut.

Composition of the IM has a direct effect on the overall metabolic health of the individual. The IM is sensitive to changes in the environment, food, stress, and other modifiable factors. Across the human population, IM differ in composition and in their ability to make SF from GRP. However, in inbred, co-housed rats, the gut communities are much more similar. The ability of the rat IM to make SF from GRP changes with the length of dietary exposure to broccoli [11]. Whereas it is known that broccoli consumption also affects the ability of many human IM to produce SF, it is well established that there is significant variation among individuals in the response of their IM to broccoli consumption [34]. For example, this might be due to absence of certain microbiota in some individuals, microbiota that enhance or disrupt SF formation and absorption. Future clinical studies will be needed to determine whether the biomarkers identified in this rat study can be useful in determining the efficiency of each subject’s IM to make SF. If any of these biomarkers reflect the variability seen in broccoli health effects within clinical studies, then the biomarker(s) may be used to normalize differences in efficacy among study subjects.

## 5. Conclusions

In summary, plasma biomarkers may be useful to monitor broccoli health benefits, potentially to normalize clinical response to broccoli. Here, we report that broccoli consumption by rats influences several metabolic pathways that impact liver health. Plasma metabolite changes have the potential to be used as biomarkers of liver health, but also to monitor broccoli benefits. Dietary broccoli caused plasma metabolite changes that correlate with (a) improved GSH status, suggesting protection from oxidative stress, and (b) the diversity and abundance of gut microbiota; suggesting that changes in the gut microbiome may contribute to the health benefit caused by dietary broccoli. Future work is needed to determine if these changes can be used to normalize findings in clinical studies of broccoli health benefits. Future studies could identify those mechanism by which specific bacterial species relate to changes in physiological function/SF bioavailability. Potentially, development of a plasma biomarker for broccoli efficacy will permit clinical studies to normalize effects of broccoli and show that, once one removes human variability in broccoli response, one can clearly see health benefits to consuming broccoli. Thus, it will be interesting to use plasma metabolomics to explore further the applicability of broccoli intake as a dietary intervention to improve health through modulation of hepatic GSH synthesis and the gut microbiome.

## Figures and Tables

**Table 1:** Plasma metabolites that changed significantly throughout the broccoli feeding.

Sample (Rat #)	1	2	3	4	5	6	7	8	9	10	11	12	13	14	15	16	17	18
Time (Days)	0	0	0	1	1	1	2	2	2	4	4	4	7	7	7	14	14	14
11-Octadecenoic acid	38.7	51.6	44.2	40.9	45	51.2	45.2	46.1	38.3	31.6	32.5	28.9	36.8	29.9	27.2	39.3	34.4	32.8
1-Monohexadecanoylglycerol	16.5	20.2	13.7	25	15.6	16	14.1	14.3	12.5	11.4	11.6	10.8	10.7	10.8	12.1	9.4	7.9	6.1
1-Monooctadecanoylglycerol	14	17.2	14.5	17.4	15.3	18.3	13.9	15.6	13.5	13.2	13.8	16	8.9	10.8	10.5	8.8	7.9	5.8
2-Octadecanoylglycerol	1.9	1.4	2	2.8	2.5	2.3	1.4	2.6	2.2	1.7	1.9	0.8	1.5	0.5	1.4	0.9	0.1	0.1
5,8,11,14-Eicosatetraenoic acid	117.2	145.2	124.4	116	96.8	104.9	106.8	105.6	101.5	91.7	102.9	107.8	107.2	95	81.3	95.4	72.1	55.4
Azelaic acid	1.3	0.6	0.8	0.6	0.5	0.5	0.7	1	1	0.1	0.3	0.1	0.4	0.4	0.1	0.1	0.1	0.5
Benzenepropanoic acid	1.1	0.6	0.6	0.6	0.8	0.5	0.6	1	0.8	0.6	0.8	1	0.8	1.2	1	0.1	0.1	0.1
Chenodeoxycholic acid	0.1	0.1	0.1	0.1	0.1	0.1	0.1	0.1	0.1	3.3	0.7	0.9	0.1	0.1	0.1	0.1	0.1	0.1
Cholesta-3,5-diene	237.8	207.4	108.9	151	127.2	154	150.3	172	123.6	124.7	120.3	163.7	125.9	124.7	77	84.4	57.3	58.7
Glutamine	1.8	6.5	2.6	33.2	18.2	20.5	18.5	44.6	27.4	30.3	25.5	37.4	66.4	52.9	47.6	50.4	17.6	2.2
Heptadecanoic acid	0.4	0.1	0.1	0.1	0.1	0.1	0.1	0.1	0.1	0.4	0.6	0.5	0.1	0.1	0.1	0.1	0.1	0.1
Inositol-1-p	7.7	11.8	5.9	9.1	6.5	6.6	5.5	8.6	9.3	10.9	13.7	15	15.2	17.1	15.6	26	25.9	8.9
Maltotriose	0.1	0.1	0.1	0.1	0.1	0.1	0.1	0.1	0.1	1.3	1.9	0.1	0.1	0.1	0.1	0.1	0.1	0.1
Nonadecanoic acid	0.1	0.1	0.1	0.1	0.1	0.1	0.1	0.1	0.1	0.1	0.9	1.1	1	0.1	0.1	1.7	1.2	0.9
Oleamide	0.1	0.1	0.4	0.1	0.3	0.1	0.1	0.1	0.6	0.1	0.2	0.1	1	0.9	2.8	0.1	0.1	0.1
Sitosterol	16.5	20	7.6	25.8	16	14.7	15.3	13.9	14.8	9.6	18.2	20.7	35.2	40.7	23.5	57.7	51.8	27
S-Methyl-L-cysteine	0.4	0.3	0.2	0.4	3.3	3.2	5.2	12.2	6.8	6	10.1	6.7	6.8	7.2	8.6	3.5	2.5	1.5
Stearic acid	306.8	360.5	271.3	296.9	247.2	264.2	278	282.6	255.1	255.9	255.5	249.1	255	230.3	208.1	227.8	231.1	223.2
Tartaric acid	0.1	0.1	0.1	5.2	3.4	0.1	0.1	0.1	0.1	0.1	0.1	0.1	0.1	0.1	0.1	0.1	0.1	0.1
Tetracosanol	0.1	0.1	0.1	0.3	0.3	0.1	0.1	0.1	0.1	0.1	0.1	0.1	0.1	0.1	0.1	0.1	0.1	0.1
Threonic acid	3.3	2.4	1.7	3.1	2.1	3.2	6.5	4.2	4.5	6.7	3.4	5.5	4.6	4.7	8.3	15	13.2	4.5

**Table 2:** Gut bacteria that changed over 14 days of broccoli feeding.

Sample (Rat #)	1	2	3	4	5	6	7	8	9	10	11	12	13	14	15	16	17	18
Time (Days)	0	0	0	1	1	1	2	2	2	4	4	4	7	7	7	14	14	14
Bacteria;Actinobacteria;Actinobacteria;Coriobacteriales;Coriobacteriaceae;*Gordonibacter*	0.00000	0.00000	0.00000	0.00000	0.00000	0.00000	0.00000	0.00004	0.00000	0.00000	0.00000	0.00000	0.00016	0.00008	0.00000	0.00057	0.00009	0.00024
Bacteria;Bacteroidetes;Bacteroidia;Bacteroidales;Porphyromonadaceae;*Tannerella*	0.04213	0.04601	0.01162	0.00223	0.00485	0.00379	0.00252	0.01583	0.01213	0.00988	0.00238	0.00443	0.00701	0.01258	0.00639	0.01220	0.01546	0.03028
Bacteria;Bacteroidetes;Bacteroidia;Bacteroidales;Rikenellaceae;*Alistipes*	0.01398	0.01098	0.01540	0.01043	0.01712	0.00923	0.00270	0.00425	0.00758	0.00204	0.00217	0.00364	0.00151	0.00833	0.00846	0.00221	0.00880	0.00302
Bacteria;Bacteroidetes;Flavobacteriia;Flavobacteriales;Flavobacteriaceae;*Lutaonella*	0.00022	0.00017	0.00023	0.00022	0.00011	0.00022	0.00004	0.00000	0.00004	0.00000	0.00004	0.00000	0.00016	0.00008	0.00044	0.00005	0.00014	0.00032
Bacteria;Firmicutes;Bacilli;Lactobacillales;Streptococcaceae;*Lactococcus*	0.00130	0.00063	0.00062	0.00011	0.00100	0.00082	0.00004	0.00016	0.00016	0.00013	0.00026	0.00011	0.00020	0.00012	0.00007	0.00009	0.00032	0.00012
Bacteria;Firmicutes;Bacilli;Lactobacillales;Streptococcaceae;*Streptococcus*	0.00022	0.00059	0.00039	0.00019	0.00054	0.00065	0.00004	0.00004	0.00004	0.00009	0.00009	0.00008	0.00004	0.00000	0.00007	0.00005	0.00005	0.00008
Bacteria;Firmicutes;Clostridia;Clostridiales;Gracilibacteraceae;*Lutispora*	0.00259	0.00130	0.00151	0.00115	0.00118	0.00000	0.00029	0.00008	0.00012	0.00035	0.00021	0.00049	0.00012	0.00045	0.00025	0.00047	0.00054	0.00060
Bacteria;Firmicutes;Clostridia;Clostridiales;Lachnospiraceae;*Anaerostipes*	0.01658	0.00799	0.01192	0.00648	0.00696	0.01793	0.00721	0.01109	0.01320	0.11612	0.02008	0.02795	0.05762	0.02817	0.01219	0.02831	0.02562	0.03390
Bacteria;Firmicutes;Clostridia;Clostridiales;Lachnospiraceae;*Blautia*	0.00000	0.00004	0.00000	0.00052	0.00039	0.00056	0.00242	0.00182	0.01459	0.01594	0.00434	0.01174	0.00785	0.02475	0.00073	0.00334	0.00041	0.00511
Bacteria;Firmicutes;Clostridia;Clostridiales;Lachnospiraceae;*Dorea*	0.00179	0.00055	0.00096	0.00015	0.00389	0.00203	0.00231	0.00113	0.00057	0.00022	0.00009	0.00080	0.00291	0.00544	0.00290	0.00250	0.00553	0.00298
Bacteria;Firmicutes;Clostridia;Clostridiales;Ruminococcaceae;*Anaerotruncus*	0.00773	0.00622	0.00552	0.00454	0.00300	0.00496	0.00508	0.00628	0.00529	0.00403	0.00345	0.00417	0.00458	0.00561	0.00501	0.00480	0.00744	0.00709
Bacteria;Firmicutes;Clostridia;Clostridiales;Ruminococcaceae;*Butyricicoccus*	0.00634	0.00509	0.00201	0.00521	0.00278	0.00625	0.00523	0.00211	0.00221	0.00100	0.00119	0.00110	0.00199	0.00334	0.00076	0.00014	0.00073	0.00246
Bacteria;Firmicutes;Clostridia;Clostridiales;Ruminococcaceae;*Cellulosibacter*	0.00701	0.00488	0.00513	0.01426	0.00927	0.01440	0.01049	0.00340	0.00635	0.00308	0.00208	0.00159	0.00143	0.00210	0.00330	0.00207	0.00195	0.00085
Bacteria;Firmicutes;Clostridia;Clostridiales;Ruminococcaceae;*Ethanoligenens*	0.00791	0.00358	0.00139	0.00190	0.00132	0.00491	0.01248	0.00753	0.00090	0.01716	0.00608	0.00072	0.02180	0.01089	0.00693	0.01319	0.02852	0.04022
Bacteria;Firmicutes;Clostridia;Clostridiales;Ruminococcaceae;*Oscillibacter*	0.02015	0.02721	0.04581	0.00957	0.01455	0.02530	0.02095	0.03862	0.02172	0.04541	0.03421	0.03314	0.04610	0.06702	0.08965	0.06104	0.04217	0.04199
Bacteria;Firmicutes;Clostridia;Clostridiales;Ruminococcaceae;*Papillibacter*	0.09673	0.13156	0.12280	0.07307	0.07844	0.00263	0.02384	0.00822	0.06857	0.00368	0.00655	0.02019	0.00327	0.00532	0.01201	0.04371	0.00290	0.01546
Bacteria;Proteobacteria;Deltaproteobacteria;Bdellovibrionales;Bdellovibrionaceae;*Vampirovibrio*	0.00398	0.00265	0.00513	0.00007	0.00221	0.00026	0.00126	0.00000	0.00164	0.00026	0.00009	0.00019	0.00008	0.00008	0.00011	0.00108	0.00113	0.00016

## Supplementary Materials

The following are available online at https://www.mdpi.com/2072-6643/12/9/2514/s1, Table S1: All metabolites detected in the GC/MS analysis.
